# Case Report: Heavy metal poisoning and acute liver failure following use of a folk remedy in a child with a burn

**DOI:** 10.3389/fped.2025.1531744

**Published:** 2025-05-13

**Authors:** Bei-bei Niu, Jing-jing Xu, Wen-wen Jiang, Xia Lin, Ling-dong Zhu

**Affiliations:** ^1^ Department of Plastic and Burn Surgery, Children’s Hospital Affiliated to Shandong University (Jinan Children’s Hospital), Jinan, Shandong, China; ^2^ Department of Pediatric Intensive Care Unit, Children’s Hospital Affiliated to Shandong University (Jinan Children’s Hospital), Jinan, Shandong, China

**Keywords:** folk remedy, burn, heavy metal posioning, liver failure, surgery, plasma exchange (PE)

## Abstract

**Introducion:**

Folk remedies for the treatment of burns are widely used in China, often due to the belief that they can prevent scarring. However, these remedies may contain complex and unregulated ingredients that can worsen wound conditions, increase infection risk, and lead to systemic heavy metal poisoning.

**Methods:**

We report the case of a 1-year-and-8-month-old boy with a burn injury who received treatment with a folk remedy. This treatment led to worsening wound edema and hospital admission. The child developed acute liver failure, requiring plasma exchange and surgical debridement. Laboratory analyses of blood, urine, wound samples, and the folk remedy identified high levels of heavy metals, including chromium, nickel, tin, and lead.

**Results:**

The toxicological risks associated with the heavy metal content in folk remedies significantly contributed to the patient's condition. Following standardized medical treatment, liver enzyme levels and coagulation function returned to normal, and the child made a full recovery after 37 days.

**Conclusion:**

This case highlights the serious health risks associated with the use of folk remedies in burn management, particularly the potential for wound aggravation and systemic heavy metal poisoning. Clinicians should maintain a high index of suspicion for heavy metal toxicity and apply early, targeted interventions. Furthermore, the optimization of treatment experience for heavy metal poisoning is crucial to improving outcomes for children affected by such exposure, and providing valuable insights for future cases.

## Introduction

Folk remedies, also referred to as ancestral secret formulas, play a unique and significant role in the treatment of burns in China. Many Chinese families firmly believe that these remedies, passed down through generations, have miraculous effects-particularly in preventing scarring. Despite widespread efforts to promote public awareness of proper burn first aid, approximately 20% of children with burn injures are still treated with folk remedies rather than standardized medical care (1).

The complex and potentially toxic nature of these remedies often results in secondary injuries associated with burns, including fever, increased wound severity, and infection (2, 3). This case report aims to alert clinicians to the risk of systemic heavy metal poisoning due to the transdermal absorption of these remedies through damaged skin, in addition to local wound complications. This often-overlooked aspect of burn management warrants greater attention to ensure comprehensive and effective care.

## Case report

A 1-year-and-8-month-old boy sustained a burn injury to his left lower limb from an electric heating pot four days before admission. His parents applied a local folk remedy for treatment, but the wound showed no improvement and instead became increasingly red and swollen within six hours. Apart from left lower limb swelling, the child exhibited no other symptoms such as fever. He was subsequently admitted to Jinan Central Hospital.

Initial treatment consisted of cefalosporin antibiotics and a 120 ml plasma transfusion over three days. However, the child's condition did not improve. He experienced recurrent fever, with a maximum temperature of 39.3°C. The fever was not accompanied by respiratory or gastrointestinal symptoms, convulsions, altered consciousness, or other complaints. On physical examination, the child was conscious but exhibited reduced responsiveness. There were three scattered ecchymotic lesions on the face, and extensive black eschar was noted on the burn wound of the left lower limb, with marked swelling. Capillary refill time was normal, and there were no signs of cyanosis or nail bed pallor. Cardiopulmonary and abdominal examinations were unremarkable, with no hepatosplenomegaly. Neurological examination revealed no abnormalities, including no hyperreflexia and a negative Babinski sign. Laboratory investigations revealed markedly elevated liver enzymes: alanine transaminase (ALT) at 3,890 U/L, aspartate transaminase (AST) at 3,208 U/L, and lactate dehydrogenase (LDH) at 2,462 U/L. Coagulation tests showed severe dysfunction, with a prothrombin time (PT) of 92.7 s, international normalized ratio (INR) of 8.0, activated partial thromboplastin time (APTT) of 296.4 s, and fibrinogen level of 1.31 g/L. Based on clinical findings, a diagnosis of acute liver failure was made, and the child was urgently transferred to the pediatric intensive care unit (PICU) of our hospital for further treatment.

**Figure 1:**
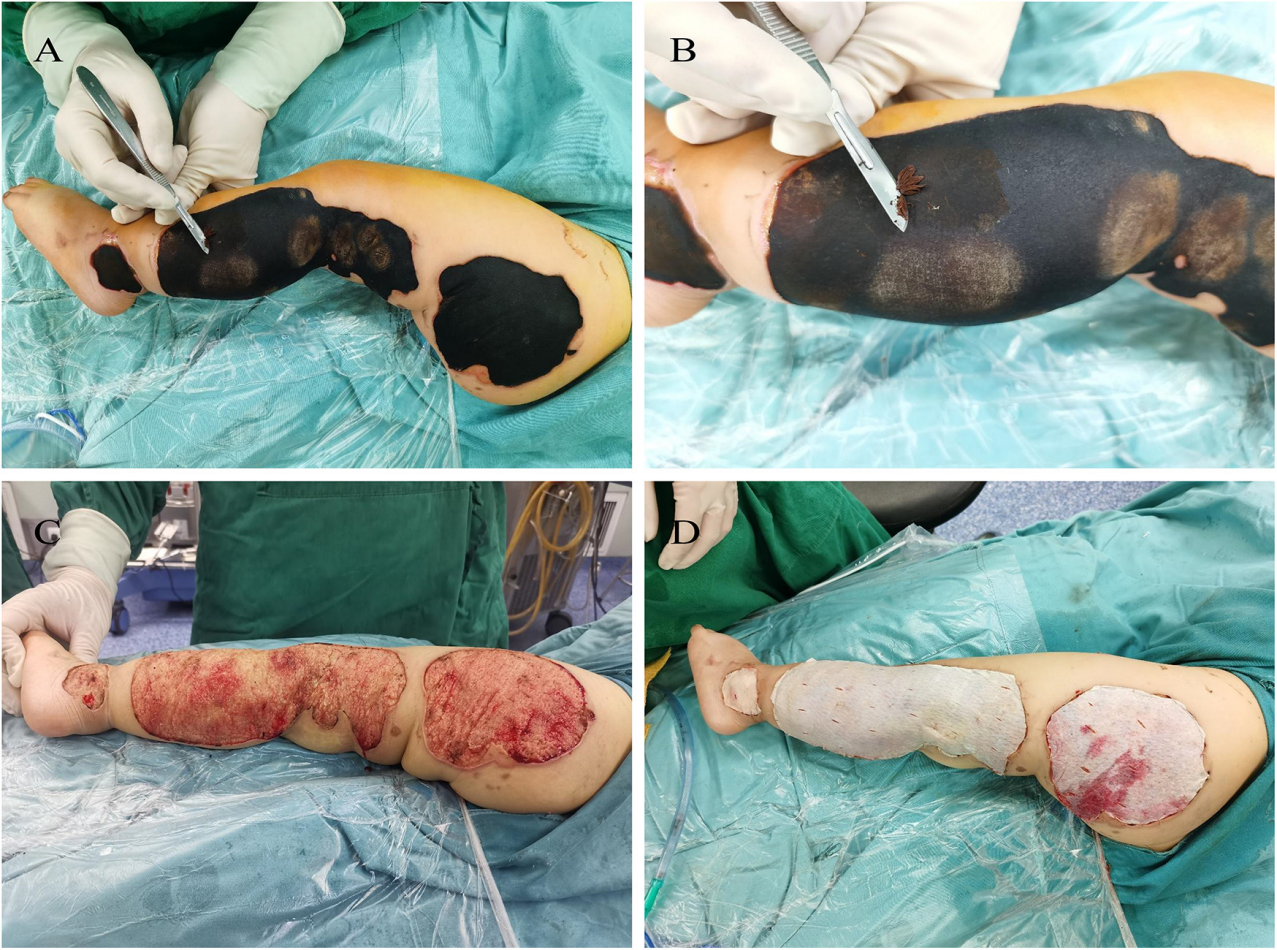
The patient's first operation. **(A,B)** Preoperative examination revealed necrosis of the skin and soft tissue in the left lower limb, presenting as dry gangrene with a thick layer of black, powdery necrotic tissue on the surface. **(C)** Upon removal of the necrotic tissue, the underlying fatty layer was exposed. **(D)** The wound was subsequently covered with an acellular dermal matrix (ADM) for further management and healing.

Upon transfer, the child appeared lethargic, and the burn wound on the left lower limb was covered with black eschar (Figure 1). Follow-up laboratory tests showed worsening liver dysfunction, with blood ammonia levels at 231 μmol/L, ALT at 6,529 U/L, AST at 4,348 U/L, LDH at 2,784 U/L, and fibrinogen decreased to 0.95 g/L-consistent with severe liver failure. Treatment included liver-protective agents (ammonium glycyrrhizinate and cysteine), plasma exchange (PE), and fibrinogen supplementation to correct coagulopathy. During this period, the child experienced recurrent hypokalemia and hyperammonemia. Potassium chloride was administered to manage hypokalemia, and appropriate measures were taken to reduce ammonia levels. Once coagulation parameters improved significantly, surgical debridement of the eschar was performed to reduce the risk of ongoing toxin absorption from the wound.

Samples of urine, blood, and topical agents from the wound were collected preoperatively, intraoperatively, and postoperatively for toxicological analysis. The results revealed the presence of several heavy metals, including chromium (Cr), nickel (Ni), tin (Sn), lead (Pb), and mercury (Hg). Notably, both blood and urine showed elevated concentrations of Ni and Sn. The composition of heavy metals matched that of the applied folk remedy, strongly suggesting it as the source of toxicity and the likely cause of the child's acute liver failure. Due to limited sample availability and urgent clinical circumstances, only heavy metal screening was conducted on the folk remedy. Other chemical constituents were not analyzed in detail (Table 1).

**Table 1:** Detection of metallic elements in blood, urine, and tissue.

Test element	Chromium	Nickel	Arsenic	Cadmium	Tin	Thallium	Lead	Mercury
Black eschar (mg/kg)	554.28	612.90	<2.0	<2.0	190.60	<0.1	79.33	<0.5
Folk Prescription (mg/kg)	216.29	443.44	<2.0	<2.0	25.79	<0.1	408.77	0.50
Urine sample 1 day before surgery (mg/L)	<2.0	148.02	<2.0	<2.0	3.76	<0.3	<1.0	<0.5
Intraoperative urine sample (mg/L)	132.84	137.79	<2.0	<2.0	30.46	<0.3	<1.0	<0.5
Urine sample 1 day after surgery (mg/L)	<2.0	17.83	<2.0	<2.0	460.35	<0.3	<1.0	<0.5
Urine sample 3 days after surgery (mg/L)	<2.0	75.81	<2.0	<2.0	169.46	<0.3	<1.0	<0.5
Blood sample 1 day after surgery (mg/L)	<2.0	273.81	<2.0	<2.0	18.32	<0.3	14.19	<0.5
Blood samples 3 days after surgery (mg/L)	<2.0	44.22	<2.0	<2.0	13.90	<0.3	12.93	<0.5

Blood samples 1 day before surgery could not be detected due to coagulation.

Blood tests on postoperative days 1 and 3 demonstrated significant reductions in the levels of Ni, Sn, and lead. Subsequently, blood potassium levels stabilized, and liver enzyme levels gradually normalized. Serial laboratory data are summarized in Table 2, demonstrating the dynamic changes in liver function, coagulation profile, blood ammonia, and serum potassium throughout the course of hospitalization and treatment. The child was discharged after 37 days of hospitalization in stable condition. A two-year follow-up was conducted to evaluate physical growth, developmental milestones, and cognitive function. Results indicated normal height, weight, and neurodevelopmental status, with no apparent differences compared to children of the same age.

**Table 2:** Serial laboratory parameters during hospitalization.

Date	LDH (U/L)	ALT (U/L)	AST (U/L)	Ammonia (μmol/L)	PT (s)	APTT (s)	INR	Fibrinogen (g/L)	K + (mmol/L)	Key clinical events
Jan 4	2,462	3,208	3,890	–	92.7	296	8.00	1.31	–	–
Jan 5	2,784	6,529	4,348	231	69.3	82.5	7.14	0.95	3.8	Admission
Jan 6	726	1,752	886	117	31.7	180	2.99	1.54	3.2	After 1st PE
Jan 7	500	722	350	–	28.1	58	2.38	0.77	3.6	After 2nd PE
Jan 8	249	322	92	–	19.4	39.7	1.63	2.00	2.8	Postoperative day 1
Jan 9	300	191	60	–	17.7	42.4	1.57	1.28	3.7	After 3rd PE
Jan 12	238	151	55	50	15.8	39.4	1.38	1.24	4.0	Postoperative day 5
Jan 16	246	55	40	39	14.3	35.8	1.24	1.7	4.6	Recovery phase
Feb 11	214	20	46	68	12.0	27.9	1.02	2.24	4.4	Discharge

## Discussion

In children, the dermis is thinner and the vascular network is more abundant, resulting in increased skin permeability and a larger surface area of absorption. This absorption is further enhanced when the skin is compromised, rendering children more vulnerable to toxin absorption, particularly from topical remedies. Furthermore, as hepatic and renal functions are still immature in young children, their ability to metabolize and eliminate toxins is reduced, increasing the risk of systemic toxicity. Therefore, special caution should be exercised regarding drug toxicity and adverse effects when applying topical agents to the wounds of children with burns.

This case highlights the risks of heavy metal poisoning associated with folk remedies used in burn treatment, emphasizing their complex and unpredictable composition-especially the presence of toxic metals. The toxic effects of these substances should not be underestimated. Clinicians should maintain a high index of suspicion for heavy metal poisoning to prevent irreversible damage to the liver, kidneys, and nervous system. Early recognition and intervention are essential to preventing acute toxicity associated with heavy metal.

Studies have shown that heavy metals accumulate in the liver in a specific order, typically Pb > cadmium (Cd) >Ni > Cr (4). When the concentration of heavy metals exceeds physiological thresholds, they pose a significant health risk. Concurrent exposure to multiple heavy metals-such as lead, Cr, arsenic (As), mercury (Hg), Ni, and Cd-has been associated with hepatotoxicity. In the case presented, heavy metals including Cr, Ni, Sn, lead, and Hg were detected in the wound, with their composition matching that of the applied topical remedy. Elevated concentrations of Ni and Sn in blood and urine samples suggest that these metals may have been the principal contributors to systemic toxicity.

The hepatotoxic effects of Ni primarily involve the disruption of biochemical processes, leading to systemic toxicity. Ni can compromise cell membrane integrity, reflected in elevated serum levels of alkaline phosphatase (ALP), ALT, AST, and LDH. Histopathological findings in experimental models include hepatic necrosis and inflammatory cell infiltration (5, 6). These are consistent with the clinical manifestations observed in this case. The patient's liver function tests showed significant elevations in ALP, ALT, AST, and LDH. Histopathological examination of skin tissue from the affected left lower limb revealed superficial dermal collagen fiber degeneration and necrosis, accompanied by bacterial infiltration (both rod-shaped and cocci) within the necrotic zones and around hair follicles. In addition, there was marked infiltration of neutrophils and histiocytes in the deep dermis and subcutaneous fat, providing further evidence of inflammation and tissue injury likely caused by heavy metal toxicity.

Sn poisoning can manifest with clinical symptoms such as persistent hypokalemia, liver injury, hyperammonemia, and metabolic acidosis; in severe cases, it may lead to toxic encephalopathy (7, 8). In this child, recurrent episodes of hypokalemia and hyperammonemia were observed following admission. Through comprehensive management—including potassium chloride supplementation, PE, and surgical debridemen—the patient's serum potassium levels gradually stabilized, indicating that the treatment regimen was effective in mitigating Sn toxicity.

Lead is a well-established neurotoxin, with chronic exposure particularly detrimental in children. According to the Centers for Disease Control and Prevention (CDC), a blood lead level of ≥3.5 μg/dl in children is considered elevated. Although the patient's blood lead level was detectable in this case, it remained within the normal range (9). Nevertheless, even low-level exposure may impair growth and neurodevelopment, including cognition, behavior, hearing, and language skills. At the two-year follow-up, the child's growth parameters and cognitive development were within normal limits, with no apparent deficits compared to age-matched peers. However, long-term long-term monitoring remains essential to assess for latent toxic effects.

Cr is both an essential trace element and a potential toxicant. Cr(III) plays a critical role in the metabolism of proteins, lipids, and carbohydrates (10); however, Cr(VI) can induce DNA modifications and disrupt cellular regulatory processes, ultimately leading to hepatic injury (11). In this case, elevated Cr levels were detected in both the affected skin and the applied folk remedy. Although a transient increase in urinary Cr was observed intraoperatively, Cr was undetectable in the patient's preoperative and postoperative urine and blood samples. We speculate that the folk remedy led to localized Cr deposition within the wound; however, whether systemic absorption occurred and contributed to hepatic injury remains to be determined.

Although As, Cd, Hg, and thallium were not detected in this study, their potential health risks warrant attention. These metals accumulate in critical organs (e.g., liver, kidneys, lungs) and induce toxicity through shared mechanisms, including oxidative stress, glutathione depletion, and mitochondrial dysfunction (12). For instance, Cd and Cr cross the placenta, posing developmental risks, while Hg disrupts cellular membranes and sulfhydryl-dependent enzymes, potentially exacerbating hepatocellular injury (13). Thallium, a potent neurotoxin, mimics potassium to impair mitochondrial integrity and apoptosis regulation. Despite these risks, the exact molecular pathways of thallium toxicity remain poorly characterized, necessitating further mechanistic studies (14, 15).

One limitation of this study is the lack of a complete compositional analysis of the folk remedy, which could have provided more insight into the specific agents responsible for toxicity. However, the detection of multiple heavy metals strongly suggests a potential link between the remedy and the observed liver injury.

While clinical reports on acute liver injury caused by folk remedies—particularly those involving heavy metal poisoning and chelation therapy—remain scarce, our case highlights the potential severity of such toxicity in children (16). Existing literature on pediatric liver failure, however, supports the effectiveness of PE and PICU-based comprehensive treatment (17, 18). PE serves as a critical adjunctive therapy by removing both free and albumin-bound toxins, including aromatic amino acids, ammonia, endotoxins, indoles, and phenols, thereby aiding hepatic recovery (19). In this case, the temporal association between folk remedy application and symptom onset, along with the exclusion of alternative causes, strongly suggested a direct toxic etiology. The timely initiation of PE, combined with aggressive supportive care and surgical debridement, contributed to the restoration of coagulation function and elimination of the toxic source, culminating in a favorable outcome. This experience underscores the need for heightened awareness of folk remedy-related hepatotoxicity and the importance of early intervention in similar cases.

Pediatric acute liver failure is a rapidly progressing condition with a high mortality rate ranging from 24% to 53%. Early recognition is vital to prevent complications and improve outcomes (20, 21). Upon diagnosis, immediate supportive care should be initiated, regardless of the underlying etiology. This case illustrates the potential severity of heavy metal poisoning and acute liver injury associated with folk remedies, especially in children, who are particulary vulnerable. Heavy metals such as Ni and Sn can impair hepatic and systemic functions through various toxicological pathways. The patient's condition was successfully reversed through timely PE, symptomatic management, and surgical intervention.

## Conclusion

This case highlights the urgent need to raise public awareness about the potential hazards of folk remedies and encourages healthcare providers to remain vigilant regarding possible heavy metal poisoning in similar scenarios. Additionally, it underscores the importance of optimizing treatment strategies for heavy metal toxicity. Early identification and intervention can significantly improve outcomes for children and offer valuable insights for the diagnosis and management of similar cases in the future.

## Data Availability

The original contributions presented in the study are included in the article/Supplementary Material, further inquiries can be directed to the corresponding author.
